# Supplementary material for: Investigating the Smuts: Common Cues, Signaling Pathways, and the Role of MAT in Dimorphic Switching and Pathogenesis
Source: J Fungi (Basel). 2020 Dec 16;6(4):368. doi: 10.3390/jof6040368 (PMC7766764; doi:10.3390/jof6040368)
Supplement: Supplementary file 1 [file jof-06-00368-s001.pdf]

Supplementary Materials

Investigating the smuts: common cues, signaling pathways, and the role of *MAT* in dimorphic switching and pathogenesis

Teeratas Kijpornyongpan † and M. Catherine Aime *

Department of Botany and Plant Pathology, Purdue University, West Lafayette, IN 47907, USA; Teeratas.Kijpornyongpan@nrel.gov or tkijporn@purdue.edu

* Correspondence: maime@purdue.edu

† Current affiliation: National Bioenergy Center, National Renewable Energy Laboratory, Golden, CO, 80401, USA.

```
Ustma      -MSSDPNFSL ISFLECLNEI EHEFLRDKGE --N-YPVLVR KLRELQ---Q
Malgl      --MLTQEEVR QKLLEFEYTL LDALTREVDE -SY-YSHLRQ LTEIVR---R
Malsy      MPYIDQDPAR HLLIEFEQAV LDALASNFGE -AE-YERLRV MSEDVK---S
Exova      ----MAEHCL NNLSTIEQRL MNSFCYPDDQ -----EQLKI DLQQTF---H
Psegl      MSDVASWTQV ANAVQAIWTI AVSFAEQEKR -------LLR RQKDVA---D
Jamro      MSVDCLNHVI EQLLQCVKSN DVSALSTLAS --D-FHAMGQ ELRRHR---Q
Meimi      ----MLSIKK NDLQAFFENA EMEFLIALQE -NS-MQSFHA KFHASC---S
Meimi_3    ----MSLHSA NSLNALIENA ESFFLSFLKD -GL-LKNFLR EYKLAS---T
Meimi_2    ---------- ---------- ---------- ---------- ----------
Viopa      ----MASRPI ARLSDFIHEF LKAYLNDPQA ----LEIANK KLITIR---D
Psehu      --MSAPSFSI KSLLHSLNEV ENDFLEEAEG --N-SAALVN KLRVLH---Q
Tilwa      ------MLSV HDLRVSLQEI EPALLQSCIE --P-GDALQR GVDRWAAVSA
Tescy      ---MSADDLS TSLLCNLKMV SDDLLTAEDD --D-YSTICT RLTQLE---V
Tilan      -------MNT NEVRHILKEL EEAVNKESKE SNSVYNPFIT TLELLLKTAQ
Spore      -MTPPDKFGF AGLLQSLRDI ERDFLSADQD --D-YLGLVS RLEALQ---R
Cergu      MSTQQQPTL QSLLALLSQS EEELLLAASN PAA-LHKFAQ SYGAIL---K
Acain      -MMPFRLQTA KDLISFFLEA EESFLESLND IDS-LREFDL RYRAAC---E
Psean      -MPSSSTLSF SNLSTVLQEV EDGLLAKDGQ ----LAISAV KLAQWP---D

           KIPNDIANLP RDPETIQQIH QTTHRI---- RAVAQAFIRF DQKF------
           EFALQQEQGT LTNATLQLGA RVSDRL---- EQLGHILHEQ RTQM------
           TISRLFEMGQ LSQPTIRLGA SIGERI---- EQLKLVLNEH RAMM------
           GIEARAAQGA FSQKELCLVY QQATRI---- KGIAMLCSFL TETG------
           DVMLLLQKQT RLSAAVTLPD RQHEDR---- KDCGQSDLHL LNVA------
           DLQLSKAERR ALIASAHRIA IFSTGLAEIE RRNSQHLRDL ATDC------
           CLTDASLTAG ISQKDATRFS AIATRI---- RDMTKSLIVL VKEG------
           LLTESIKDKT VSENMVSKAR SALERI---- KAISEALLLL SKAC------
           ------MERK RSPRDAHRHH KGEGGV---- ---------- ----------
           EIREDLAKGT IGRKDAATGF CIAGRL---- EAITSSFIGL EESL------
           ETSEHIASIA RDPDSIRQVH NAAGRI---- QLLADTFVYL DKQF------
           SFNQLRASGA LDDSTIELAH RVAQRT---- TIMSESLCRI ERGLAGVLLD
           TARAICTQRT TDDTVADQIW QTSERI---- KIIASTFLRL DEYR------
           SVNHAEHQNG EAVPTFQPSH AAAHRVV--- EVWLHVLERL KCISEKRQAK
           KAVIDIPTCS PDQKSAAEAR QAIQRI---- QLVAQTRHRL EEMF------
           SLQSLPSLGT REHESIVTAV TRVERIAAHF RECEQEFARV RAAL------
           AAKSLAANER LSEEVATIIG ETTSRI---- KLVAETFHKL ESNV------
           KISKSKEHGG LDQASVDHLH HRVKRI---- QIVTKAWLRL EEVY------

           ------VSLC SE----VVHG TSKVMQEFN- ---------V VSPDV-----
           ------QRIH SN----ASQE LVLILRSPI- ---------P RSRAS-----
           ------DELQ KS---TSDEI QSILEKESI- ---------K LTQAF-----
           ------TRVM SH----AKEE MQGCFSAKP- ---------V EKAIFPEKMV
           ---------- ---------- ---------- ---------- ----------
           ------KELV ATSELASTKT TSTLRHHQG- ---------T GTRGS-----
           ------DMIA KE----AVEK TKAILSGRL- ---------R KEQCS-----
           ------SHIC EE----AVKD VNEVICDGH- ---------E NPEVK-----
           ---------- ---------- ---------- ---------- ----------
           ------QIIE KQAMIEQDEV FSEFVKSKR- ---------K TEKPFDSAKP
           ------VSLR AE----MIRD ASKCLQATE- ---------K VQSDP-----
           FRNGCAEILE KDNEELGARA GRASLQDRRM PSRLANSPAV TPQSVPTPAA
           ------KKIQ SG---LSSID LKKLQRDHR- ---------Y GSDTK-----
           VMAVLIRGKE APAKLSKSFS PSIQACAGV- ---------C SSSASSASAS
           ------EELD GE----MLRE ANKAIQDNA- ---------K SKQSL-----
           -DHDCEKELA EH----RASL KRLSYRKGL- ---------S ESPPHLVDVA
           ------LDLD KS----CSNR TKQILRSNT- ---------E GSDKR-KETP
           ------SQLN SD----LKEN TFQISCKQG- ---------Q HSRNT-----

           -----GC-RN LSEDLPAYHM RKHFLLTLDN PYPTQEEKET LVRLTNESTA
           ----GSLRDR RDPTLNAKHM RDWFLRHLGH PFPSREDKEQ ILAETNACIR
           -----VSSER RDAGLNAVQM RDWFLKNMGY PFPSRSDKLR ILKTTNRQGA
           EPAKSSKLNI NQDTLYSTVF REWFLDNLAD PFPCRQTKEE LVLKTNNLLE
           -----SSSKY SEQSLNAAIM RRWMLEHIDH PFPNNHDKEE LAKETNRRAP
           ----QLRSQP PDSALNATVM RQWLLEHIDH PFPSNEDKRA LADESNRRGV
           --------PT NQDSLNSQFL RRWFLEHIDH PFPSTVVKEE LAELTNAKLK
           ---------D KRERLESQPL RSWFLKHIHD PYPSAKEKEK LIKLTNANLK
           ---------- --GAVNLPFL HQWFIEHIDH PFPTQEEENE LAMKMKANLR
           EMTKPQLPDD SQDSLPATYM RQWFLQNMHD PFPSKAVKIK IIADTNATLR
           --------QD ISENLPSYYM RKHFLETLYS PYPTQEEKEA LVRITNESSA
           STTAPTAATA AELQLNAVVL RRWFRENLSS PFPTKADKTA LVTETNALAT
           ---------- LAESLPSSHL RRHFLSCLDD PYPLPEEKNE LVKSTNEYYA
           SPTSPAKWSI SSEQSANNIM RRFFIEHMNR PFPNKAEKEK MLAQARSVTP
           -----EVEQD LSETLPSYHM RKHFLATLDN PYPSQADKDT ILRTTNDSAA
           PNAAQTPLSN VDWRLNAVPL RRWYRENLYD PFVNATTKEA LVREMAAMPK
           LKSDETSEGS LSELLNAKVL RRWMLDNIAF PYPTREEKEE LARHTNLQLG
           ------GPPD LSESLPSYHM RKHFLEHLDN PYPTQGDKES LVEATNASAS

           RVGQSSV--- -----N---- -RPPLEVHQL TLWFINARRR SGWSHILKKF
           D--------- -----R---- -TLRLKYTQI VLWFINTRRR SGWTAFLRHY
           V--------- -----K---- -SQVLKYNQV VLWFINTRRR SGWTKFLRRY
           VVLLNEKMGR L----I---- -YQPVDLSQC SLWFINTRRR SNWTSFFRNF
           S--------- -----G---- -KSQLRPEQA TLWFINTRRR SGWTSWARRF
           G--------- -----T---- -KGKLQPGQC TLWFINARRR SGWTLWARRF
           DLESRDELPK FGPQSA---- -VKPISKNQC QLWFINTRRR STWTEFYREY
           E--------- -----A---- -NKPMSREQC NQWFINTRNR STWKEFFIKY
           DIETKIGLSS S----S---- -VSSRATKQC QAWFINARSK STWTEFYQKY
           TSNRS----- -----S---- -FGPLTYNQV MLWFINSRRR SGWTTMFRTY
           KDDPCST--- -----S---- -RPPLGVHQL TLWFINARRR SGWSHILKKF
           GSDAGPSSAV AIPSVGSAAA SNKTITYEQV VLWFINARRR SGWTDFFRKF
           RNPCAEG--- -----S---- -QQAMNHNSL ALWYINARRR SGWSSILRKY
           E--------- -----G---- -QKPLSASSL SLFFINTRRR SGWTEFFRKY
           GT-------- -----G---- -RPPLDAHQL TLWFINARRR SGWTHILRKF
           ---------- ---------- -AKRMNAEQC TLWFINARRR SGWTEFYRQF
```

```
PPLSMSLGES TNIGLS---- -GTPITSDQC ALWFTNGRRR SRWQEFNRTY
QRKKSEE--- -----R---- -GNDLQINQL TLWFINARRR SGWSSILRKF

AREDRSRMKH LVRA-K---- --LSSSNQST PPSSTSDS-- --LSNNLDDV
ARGDKVKLFN LAQAIE---- --NEEGGTHE TRQWSAGH-- -AITLTPASA
AQGDKTKMLE LVWALE---- --TSVGGTHA SRYWSSGIGP VSATKIRDGK
AHSDKNLMAR LVDCLK---- --REQDGIDA PE--TMGE-- -LEDLLKDEK
ANSDKARLRA IVAAIR---- --GEVLDIRG AEEEVNDS-- -LEELVEAHD
CRNEPPLTRA IVAAIQ---- --GRPILPTT KSEAGEGD-- -EVDQIRQEQ
AFSEKSTMTK LVSTLK---- --GESESANG TSKSLARI-- --LTCGEDGR
GKSDITTMKK LIAILE---- --NESEKTIW TSEALAQF-- --WSFGKRGK
AFSEKAVMTK LVSILK---- --GESENTNE TTDSLARL-- --WWCGKDGK
ANKEKNRMVE IVEALR---- --REDNGQTD GGKPLEAI-- -LTQFGGGSI
AREDRSRMKR LVRA-K---- --LNTSDLST SPSPTFGH-- --LPLTLDDI
AWSDKARMSA LVDALS---- VSASDRAVVE DATPALRP-- -LLTLSKEGR
ARNDRQLMKP LIQA-K---- --MMQEGLPV RALLSGEL-- -DTAHAVHAL
AGESHEVMSR VVQCLEEEKR RNMDDQASVG TMRTTVGI-- -SLQGLLDEI
ARGDRSRMKV LVQT-K---- --MASSGLLA PVPSGT---- --LACSLDGV
AYGEKDRMRI LIQALE---- --KDRSAESK HLHEMFAI-- -DHKGRRATF
GLNLRGRMEH IVEVIREHNE VHSNADDHST PCPALEDI-- -LKKDAKGGV
ARNDRQRMSL LMQT-K---- --MDACNMSS QHERSPAI-- --LVHSVDDV

LSDNLG---- ---------- ---RPLTPVD K--------- -------QQF
VQANKPMLSD PNTSSGLSLQ SLLPNMDDIA R--------- -------RAM
KERKIT---- -----RMTIK EYMPNANDYE L--------- -------EKM
IDTISC---- ---------- ---PITLAAR L--------- -------KKC
VKDRGL---- ---------- ----TKTELA ---------- -------ELC
QPDRLE---- ---------- DLIDPADDVE K--------G LNPTQLAELC
TREWFG---- ---------- ---ESLKKRV ---------- -------AMC
TEEWFD---- ---------- ---GTLRKRS ---------- -------ISC
TRGWFG---- ---------- ---DSLKEHV ---------- -------AKC
SAKQKA---- ---------- ---------- ---------- -------DKC
LRDNLG---- ---------- ---RPLTSAD K--------- -------KVF
ARKKAD---- ---------- ------IEAD L--------- -------VAC
LRKNLG---- ---------- ----KVNDKI Q--------- -------KDF
QSGKTARKKP RKNSSAGSGT VQSPSPMAIR KTTRSTSTSA SEPKLDAETC
LSDNLG---- ---------- ---RPLTAAD K--------- -------KEF
MKVNKD---- ---------- ---------- V--------- -------KDC
VNKSKG---- ---------- ----DIAKMS ---------- -------SEC
LRDNLG---- ---------- ----RLTDAD K--------- -------KEF

EDDWASMISW IKYGVKEKVG DWVYDLCAAS KKTP--KPGM PR--------
KREWSNIVDR VRIGAKERIG DWVDEVISAP STAS--SSSQ SR--------
YSEWKKIVER IRYGVKERVG DWVEEVIGTS SDL------- ----------
REAFTTVTDW LRHISREKVA DWMDNIVQEA KVEL--QVEK AT--------
KAELLKVTDW IEQGSKERVG DWMDKVVSEA KATL--KRKK QD--------
RHEFEQVIDW VEQGANQRVG DWMERLAEET KGVA--VAKK GK--------
QDHWVKIMEW LEQRPHEQQS DWLSQAIEEA TSEY--NQTR KS--------
WEHYSCIVAY LELRPFEKKS DWLSEAVEDA TIVY--NRMS KR--------
KEHWMKIKEW QEQPQREN-D TWQKGSKPSR KDSR--RFSE ET--------
RQEWKKTCDW IRVGVKDRVG DWMREVVGVE ADGE--DESQ RV--------
EDDWASMISW IKYGVKGKVG DWVYDLVAAN KKAP--KHGK AR--------
KAGFAKLIDW VHQTAREKVG DWLDVVLAEA AAER--QAKG LR--------
HDDFTSMISW IRYGVRDKTG DWVQDVVNAS KKKT--KHKR GA--------
RAEYEAIIEH VAVKHPERVG DWIEQVVQAA AAIRAGQPIP SALLSFSSAQ
EEDWDSMLSW IKYGVKEKVG EWVYDLVAAS KKPP--KAGQ AR--------
IAAFERMLSW IHQGGQDRVG DWLDEVLAEA AAER--AALE AQGFDSPQAH
RKLFAHVIAW VSQSTKERVG PWVEDVIKKV VAEE--KQRK KQ--------
EDDWTSMISW LKYGVKEKVG DWVYDIVANS KKPL--KAGQ PR--------

--------PV TTVAKRHPAR KTKPAAKPKS RTANPRA--- -------STT
---------- -----RHSTR RTTP------ ---------- ----------
---------- ---------- ---------- ---------- ----------
--MREQRKRL LDLEDKAEEE RDHRKRKLVP ARKKNVAKVA T------GQS
--------RR AARKLLAEQR RRRAAAATRD HKMEDSEDEL A------EEQ
--------GK SKKRAREEAQ RKALAAAPKR ARTSRAAQDK V------RIR
---------- ----KRDQHR TENGSHA--- ---------- -------HQS
---------- ----QRNLDR ETQEFKLQKA AAH------- -------RSD
------LTIL LDEYERNPHY ENHLDRDRIA KKTGLKPRQV TVWFQNRRSR
------PTAS KTKGKKIKAR PVRKGKSITS STMK------ -------TTK
--------TV NTAASRSPAR KTATATQSKP RKAKQRA--- -------SKT
------DPSK PLAPVSQPAK REETPPAAAL VTTANATRAI A------GRK
------SHIV TNAGNRSPLR KSQSCLSSSR GDPASQTRRA T------SSK
TSSTAPESRA NSVAGAATGS MAKQSASRQG SKEEAKSKKR PISQVGSVGG
--------AV TTPAKRAPAR KTATPAQSKP RKPKQRA--- -------SKT
TSVEVAPTPG PTRPMRALPT RSAGKEPAEE SRPIRGSKRA G------QRA
-------RRA GRRRLREEQK KVQEQKPAEL GRGARRLRRS SRSVAPYSTA
--------TV TTAANRTPVR KTTTSSRPKR ATRR------ -------ISK

PSIDSTLDSS KLESTPELSM CSTADTSFST FGSSLSMSHY NPFQDGNDIL
---------- ---------- ---------- ---------- ----------
---------- ---------- ---------- ---------- ----------
NSIQKRKSNE RKERVQYRKL PKVIVHTPDT SLGHIQFSDF SAGTASRSSS
VPAPARPGLR PIRRMPQQRN VSGASSSSSC LSSMISGRLY PSGSSSSAAT
ESDADDDSED ELPASP-LAG KSTKGLPRVT GTQGCTSSDY SAMSSRNVSG
DDEDYSDQLQ SKKRVKKRRT SDGHARPIRG SRRSRDSARK NVRRPSTMIS
DDDDDAEQVR SSKRAKKTNT SSTDQKTKAG NSRSRDGANT NARRSSKSSL
SENPPLKEAT EKRRKREQDK CQGGDKKRHA PIRTKSSQR- SVNQPVPASP
SGRKVKPTNK ELTIIAKQRF DYYTTSSESS GPNTATSASF DFSNEVAVQP
PSIESNTESE RFESTPELSM CSTSDTSSST LDSNLSMARY SPFDLGDDLL
TRRLPKKTTP RTPSPPVDPK ASIGASPTTT SLDGQTKLAF TLPGAAQFSL
PDGWTTPKQE REILDTSFNS CRSEDVKVPG SSTTETRFNI QMLDYLAGSE
AGGVGGAGGA GDESGKEDDD DDDGSSSSKS NNKTRDSPST SHRRPSPFFP
PSIDSVADTS GLESTPELSM CSTADTSLSS FASNLSMMQY DPFQHRDDLL
LGVNTGSTAR QAARNASSSS TESSSSSVPS LAMSQDSASS TAYSTISSAS
```

```
ARRRPVKTTG KPSTVRQNVT ENKVDAPIRT SSPSLLYTNS NVVTPQSRKF
TPSLGSDISS EHESTPELSM CSTADTSFSS FGSNISLLQY DPFQASHEAL

QSPTVKAR-- GNRKV---KA LPKRAGKQQP DEVDNGKIPF LCLSVAFV--
---------- ---------- ---------- ---------- ----------
---------- ---------- ---------- ---------- ----------
GSSYQAPSLF NMSDY---IY PPSSCASSAS SLAASLASYD AFHRPPG---
SFDSTYELRS SSRRF-ATDC KTESAGRYFQ QLQVSDDPSS VEVCSPSPT-
GSISTAPT-- SLSAS---AT LPETHKGNAA S-------RP SSSGSSLAFS
SIDQYRDV-- SGSST---ST LDTSCSSIST SSYATASSPL ASYG------
KK-------- ---------- ---------- ---------- ----------
SLPVYDDDVV ARIDRDVLQL RAIYNESFFK DLDLSSQRSS SIRQDLSPDK
KLPSHLAH-- RSQQR---DF SGSSSSTLDS AITFDTEAPS VSSRTSSTFS
KSPSLNAL-- NSRRV---KA LPKRGQKQLL DAMHTGCDSF GCSCLASEFR
RVPPPTTS-- LAGRR---DS TGSRTVSGSS TNSVGPSVPP LIGGAQDLSI
RQVKRLPSRG RRKAS---ST VPAHVHGISG TVSDAPSAFP S---------
PVPLSGLGEL HGRSDALRPP VPFFSPDFAY ATDSSYSQSH FQGDYTLETP
QSPTLHAK-- STRRV---KA LPKRATQQQQ SPRKTLSKDE SATGTCL---
SSPINKDG-- NMATLLSSQI GPANADVLQS ILGFANESQP KHFGDAASLP
SGISHASSDW SGASFDSMPS LISDCSSSFS SSFDSSVPSP TFSRSNIRP-
LSPTLQAA-- ATRKV---KA LPKRSQKADS SDEGGSLALA MQS-------

---------- ---------- ---------- ---------- ----------
---------- ---------- ---------- ---------- ----------
---------- ---------- ---------- ---------- ----------
---------- ---------- ---------- ---------- ----------
---------- ---------- ---------- ---------- ----------
T--------- ---------- ---------- -----DADGQ AAPSALSH--
---------- ---------- ---------- ---------- ----------
---------- ---------- ---------- ---------- ----------
YLLE------ ---------- ---------- ---------- ----------
SSLSGALG-- ---------- ---------- ---------- ----------
SPFRTAKFSF RHTELHLLHN LRFLSYSLDL LRFLTILPST IPHHPQRFRA
G--------- ---------- ---------- -----SSPSD ASMSSPPR--
---------- ---------- ---------- ---------- ----------
PPPPAPAPAS QDPLVHNRYY RPELVVLSQT QSGVIARTGA GDGA------
---------- ---------- ---------- ---------- ----------
NAFAASRSAI QARALNMHDG LKTPMQIDAT TLGESNVSTS VDSDSSSD--
---------- ---------- ---------- ---------- ----------
---------- ---------- ---------- ---------- ----------

---------- ---------- ---------- ---------- ----------
---------- ---------- ---------- ---------- ----------
---------- ---------- ---------- ---------- ----------
--------SP TLNIRKNSQP RELKQNELIP ALMDAQMPIT NVSSLYTSNL
--------GG LSRLPPFAPQ QQQRAEQAHR TYEMQQASLT RLQPPASSSW
--------PW VQQYLEQQRA ENAATMAAPP SP-------S NIRTATPSAR
--------KA QETLEQKKAD DLEGLYHEPK TSFINPKPVF SPIPASPLQY
---------- ---------- ---------- ---------- ----------
--------PA ALPEPTSSGS MLLSHDMLMN ELYSARSPLT YLDPDAASYF
--------PI EPNTRKLSTV KLQDSTQLEP SS-GSSETRF NPSCSPSKVW
PQTMSSLRQS PLPTLSALAS SFTDLNTLLQ PLLSIPYSEL NSALQSNSSV
--------PL SMPTFQHMIL HDSPLRRQQL AQ-------Q KQQREMLAVY
--------VL LPAGMGIQSQ QLGQYEHGPN MVTQAPVFTV TARVDPPFQS
--------SG KLAAKGLSSE RRRGLLSLDP SLQFGYIPSE ASGLTVAATS
--------PV PEDFKLPSTS AAHAQAALST QP-------- --QLFPPYDF
--------NS QERGHSISAF ETITLADVTP TKNSVRKSWL PSQTAGQFTF
--------TA PLPRSSATSA SPTPTRQQHS LTAGGQSPTL SIRSRGSSTD
--------NR LIPTPAESSL RLTSYPSAPH TLTGAQTTGV PTQLYPPYD-

---------- ---------- ---------- ---------- ----------
---------- ---------- ---------- ---------- ----------
---------- ---------- ---------- ---------- ----------
VPQLPLNSEP IPCIQGLQFL PSE------- --LD------ ----------
--SVTSGSEA VRYDQQSDGS QTF------- ---------- ----------
--DRPVRKLP ARAAAATSAM ASS---SSNV FV-------- ----------
--GDDEMGDD DGEFELDDFE EAK---QGDS PWN------- ----------
---------- ---------- ---------- ---------- ----------
--EATIHQDL SPDMYLSNFI RLDRR----- ---------- ----------
--KYVYRDEN ASGLERSSFV TPL---VEHT LPST------ ----------
--KSNTEDEL QGRLDAARMQ VSVAYVLMDL VWILLKTKGV DTRDHPVMQE
--KNESHPSC SPGSDASSGV SIS---ELST FPSTGSQDHS MQSSQSSHTL
--TLSAALG- ---------- ---------- ---------- ----------
SPNHTSLNGV ADAAAVAVAV AADNKQRRHP HGHAQSMHRQ PSPPAPVSNF
--ALGQAPMP IPNIRRESLS SNS---LSAA FG-------- ----------
--GIGAATTP ANASASTDQP SSA---QDAA TTWEPFSFAI PAPSASAQAS
N-DYEMMGEG EQQHFPASFT TSNGHMHGRQ MTFS------ ----------
----ALGRAP MPAIRRDSLT SNS---LSAA FC-------- ----------

---------- ---------- ---------- ---------- ----------
---------- ---------- ---------- ---------- ----------
---------- ---------- ---------- ---------- ----------
---------- ---------- ---------- METELSGGTY SQLTNIQPQA
---------- ---------- -----FMPSV TAAAALGNNW GMPVCTSAVD
---------- ---------- -------PPT TAAAAVGANW GLPPTSSGSG
---------- ---------- ---------- ---------- ----------
---------- ---------- ---------- ---------- ----------
---------- ---------- ---------- ---------- ----------
---------- ---------- -------GLI SNSEGNPHEL GTECRGPSTW
LERVKSYFAK IKGVQEKGKI EEEKGAKVDK SAAGRFIRAA LAGEGGAVAG
RPVDSSPDYN AFICASTTPS PFPDKPTPAS TSARASPDDS GFFENEAGVW
---------- ---------- ---------- ---------- ----------
DLFLDSTFHL GVDMGHGLGS WTSASGSPTT RSAALLPAPS SSATSSMTQY
---------- ---------- ---------- ---------- ----------
PSKTKAA--- ---------- --------ES ATDTTEPFAL GTLASTLDTE
```

```
---------- ---------- ---------T LGSSPLPLAA PRSRQGQSEW
---------- ---------- ---------- ---------- ----------

---------- ---------- ---------- ---------- ----------
---------- ---------- ---------- ---------- ----------
---------- ---------- ---------- ---------- ----------
IDASSLIDQY STFQGW---- ---------- ---------- ----------
HSVTGQSDDA VNYGGWLAWS GQMMTGEA-- ---------- ----------
RSPGVITIPE WSWTSRHN-- ---------- ---------- ----------
---------- ---------- ---------- ---------- ----------
---------- ---------- ---------- ---------- ----------
---------- ---------- ---------- ---------- ----------
EPFKHEFDNE KEKNSTNECG KCFASWPS-- ---------- ----------
KHTKFDEETP VERNGKVEKT ETSKKEVAKP AASTPASGKK KRPAMDPFEG
DPFPLSQHLA IDSHRAHGAF GSLVLHDAPS QESIIWRTPE HSQLEHAEET
---------- ---------- ---------- ---------- ----------
GHSQGLSLNL AHDQHGHGHS SGLLFMHPSR AFDGAVSTGS GASMHSPYHG
---------- ---------- ---------- ---------- ----------
ETNEADFLTL EFLNSVTEAN AQDKEIASWA VYNSDWPTPG LAGLSDFEFL
HGNGRSDFAP STAAARSHSN SFSSSSTSSS TTG------- ----------
---------- ---------- ---------- ---------- ----------

---------- ---------- ---------- ---------
---------- ---------- ---------- ---------
---------- ---------- ---------- ---------
---------- ---------- ---------- ---------
---------- ---------- ---------- ---------
---------- ---------- ---------- ---------
---------- ---------- ---------- ---------
---------- ---------- ---------- ---------
---------- ---------- ---------- ---------
---------- ---------- ---------- ---------
YDKPKTPKTE SAPTTKSTDS PASSPSKKQK KSKKSSAK-
RKTMRRSPVD EALTLLAAPS LSRNASQNSH MSS------
---------- ---------- ---------- ---------
MLFGD----- ---------- ---------- ---------
---------- ---------- ---------- ---------
EALATQQ--- ---------- ---------- ---------
---------- ---------- ---------- ---------
---------- ---------- ---------- ---------
```

Figure S1 Alignment of bE (HD1) protein sequences. The bE protein sequence from *Ustilago maydis* (UMAG_00577) was used as a reference sequence to determine orthologs in all 16 Ustilaginomycotina genomes. Then, all orthologous sequences were used for the alignment using the MUSCLE algorithm performed in MEGA-X. Note that there are two duplicated *bE* genes in *Meira miltonrushii*. Species abbreviations are as follows: Acain, *Acaromyces ingoldii*; Cergu, *Ceraceosorus guamensis*; Exova, *Exobasidium vaccinii*; Jamro, *Jaminaea rosea*; Malgl, *Malassezia globosa*; Malsy, *Malassezia sympodialis*; Meimi, *Meira miltonrushii*; Psean, *Pseudozyma antarctica*; Psehu, *Pseudozyma hubeiensis*; Psegl, *Pseudomicrostroma glucosiphilum*; Spore, *Sporisorium reilianum*; Tescy, *Testicularia cyperi*; Tilan, *Tilletiaria anomala*; Tilwa, *Tilletiopsis washingtonensis*; Ustma, *Ustilago maydis*; Viopa, *Violaceomyces palustris*.

```
Ustma      ---------- --MTLPP--- ---------- ---------- --------LP
Malgl      ---------- ----MHPELD FLDECLCTLR H--------- --------VR
Malsy      ---------- ----MSQELG FVEACLSMLK SL-------- --------SA
Exova      ---------- -------MTA SDLLCLESIA STAETCLKVL A-------LH
Psegl      ---------- --MSHLSLLE DYLSCSNRIL DVLSKDC--- --------LL
Jamro      -MSCKKERRG ANVRFDEDVT ARPQAQGPFV PFAPTPLGPR LSRGTQTRQP
Meimi      ---------- ---MVDSSKQ NDLREIIKVS NALMQQ---- --------FD
Viopa      ---------- --MMSDRFSL QSLRKIQEIS SLFK------ --------VK
Psehu      MRLPLFPSSS IRNAFSRPYT NMLKETMTDV DFFSQVISLT SQISTN--LT
Tescy      ---------- ---------- ---------- ---------- ----------
Tilwa      ---------- ----MTSARL DILAEIHRLS TLVHHTVGRS LGQCSERDTP
Tilan      ---------- ----MLNEST AIIRLLKELR EALAQSAEHD NLQPLDNQEH
Spore      ---------- ----MTAATV STLTQVHALI TEIISS---- --------LP
Cergu      ---------- ---MVEGCLS DSLKAINKTA HAMLR----- --------VM
Psean      ---------- ---------- ---------- ---------- ----------
Acain      ---------- ----MPFSLC NFLEEVVRAA SIFDD----- --------LP

           RISQTAPRPT CFLP----LS LEGPNQQA-- ---------- ----------
           GTSTPVLPSP VMDAPDAQLD LQSVHSQS-- ---------- ----------
           SDETLLPSPG IYSPLAE-LN LRDINAVS-- ---------- ----------
           HTSEKTDTPL NARQNTL-LN LPDVDSQS-- ---------- ----------
           PVQQDMNTST VTVPH---LP KQGTFAAE-- ---------- ----------
           PSHLPSPRYP SFQMTDLQHI LRGIADRVNV FLEEFDVAGV ATAAPSSSKL
           ITDACSSEKE DKGSWDPPKR LSYPLVNA-- ---------- ----------
           IGSTILPTEA SKFPVDP-LY LNSDGTYC-- ---------- ----------
           TASSPIRKTV KLTATIP-LN LGYVEEDS-- ---------- ----------
           ---------- ---------- ---------- ---------- ----------
           LGPRSQPASQ SFQRSL--TL LRDEAIAS-- ---------- ----------
           MGHLPSRRTS TANIWASQLS LAKKD----- ---------- ----------
           KAAEPVHCSP PFVRLDL-LN LMSPPSEV-- ---------- ----------
           GAEPLASTSD RISPSLRPLL LDAPRDEL-- ---------- ----------
           ---------- ---------- ---------- ---------- ----------
           TSFQPQPSTS SNSGAT--FD LPHCDVRS-- ---------- ----------

           ---------- ----LSR-KL SKLGIGSVCR DTLEEIFIEY LRKLRRVYEA
           ---------- ----FVA-AL RLRPVSNDVY DALIDLYERV LEQLKRMYMD
           ---------- ----FMS-AI RERPLSNDAQ VLLIRLYERV LIKLKGASME
           ---------- ----LHQ-EL SKGGITSSTL LKVLQFYQSE AQIARENFNL
           ---------- ----LRA-RL HSRALGETLI ESICDLYQSR CCIWQEKIQV
           NEDLIPPISW FTGRLQS-LL KALPLDESLR QQIINIFTEL GQARRLQVLE
           ---------- ----FDR-SF RHLNLSLHMK RRYEEKFTEV SDQLHSLCLA
           ---------- ----LQE-EL GSRPLRPETR FLITKAFEKG ACDLQKTFRT
           ---------- ----FSL-DL SGLAVDKAYH DALLMLFKNQ LVDLEEAYQI
           ---------- ---------- ---------- ---------- ----------
           ---------- ----LET-DL SERPLRADVR SSLVARFSVA CDESLSAVQR
           ---------- ----LQS-RL LQTRLQPHEI SELLRVFSAH AADAITSLTI
           ---------- ----ILP-DL LIFKLPEGCQ EALIGLYHSQ MEALRVVYLD
           ---------- ----LSGFNL KLYPISPSAR NALEEKYVEM CNVWTRQANA
           ---------- ---------- -MLSLIFSRR DKPESPFSKL SVSLSSANSF
           ---------- ----FRS-RL AQRSISSDAS ERLHNLYYRG LQQFRAEYVE

           QYENAFVTWQ QENLY---EE AYDQAFRKLL NRLFAMHSQE TWHMVLDEVS
           HFHDAQMRWG SA------RQ RVLPQIQRLF EVQCQMAARD MQKAILSLVD
           KFTQIQRRWG SA------RR RALPRMEQLF KLQCQLAATR MQDALLGIID
           HWHANFDSFI SAQ-----DE EFVKALQDVF RQRFIEHLTS LRDRLVAVIS
           EWIKSYTRLV GLGG----KA STLRLMQKAC IANLSRGMQC MQDDVLSLID
           RATSALQSLV SLEG----TS EQQDLYIKAL QSQYSRRTME CIQVLYDSLK
           EVVKIASSLH GKL-----HN CDFEKIVRIT ETSLSENVSQ LHSSMMATIQ
           QHETLTCGLQ SLFVDEDERV FKLDLVKKCL EARFHGAIEE LLKIIVSMID
           EYDKAVTVMR QENLY---EE AHDEAFRGLL TRLFATDSQR VWHVILEEVR
           ---------- ---------- ---------- ---------- ----MIDLMK
           GRATALHETS LP------SA QYLAQVEQAL LQSLSRSLCS NRQTLLRVVD
           ADQAMYRVLY PSP-----AA SARSPHETSL RFLYQQWLDQ EVSTIMATVR
           TYKKSATSLQ YHGQP---DE TFFTSFRTTL EHNFSTQAGT MWNYLLEEVH
           HYLEAYYRLT ALSTP---EA SYLESMQAAF RRKHAQGLSR MQDVLIQCIR
           SIASEFGRLI SDG------- ---------- ---------- ----------
           KTAKLLQALP KQH-----EQ SSAANIEVAL TRVYLNNVAN LQRAILVEVD

           K---VFRTDS SLTVTQR--D NASYEGAPL- -KTGRGHDSE AVRILEQAFK
           DRLKTFAADA NAPH------ ---------- -ETHRPHSVR ATAILERAFA
           ERLKNFQDAA NEPT------ ---------- -DAHRGHSPK AVAILEKAFE
           QKLALFWRDA EASAQSE--S ESCSEDGL-- -FIPRGHSRL AIMILEKAYT
           ERIEKYRIDS EAAASAS--S GDQFSDSDDD -DNARGHSAE AVAILEAAYA
           KRFVAYQVDV DSAASTA--E SSSESDDGD- -GCPRGHRPA AVMILERAYA
           NKIKAFHADA AFSDVES--S GESS------ -VAQKGHSRR AVAILEKVFS
           ERIAQYEQEV ASSDSLQ--E DDE------- -QPGRGHRST AIAILEKAFE
           RAPLAIARQS KPVSTGA--G CLVFSDGETH -KVGRGHDSD AVRILEQAFE
           QRQEMFHQGT FQGL------ ---------- -SEYAGHNQR AVSILEQAYQ
           DAVARFRSDA EAAAAASPFG DESEYGGPQQ PEDARSHRSA AVAILEKAFE
           QNMCTKPFSH IS-------- ---------- -SSRRLHEES AIQILEYAFS
           RFTGAGHPFK AGPYDFG--S LASSKEAQS- -KTARGHDSD AIRILEQAFE
           DGVTRFNNDL FPEHKHD--S ATSNLDTAH- -GVSHGHSTA ATALLEKAFE
           ---------- ---------- ---------- -KANRGHDSD AVQILEQAFQ
           TAIARFHADA EASAQSE--G ESSSSSSEDG LNVSRGLPPL ATKMFEAVYA

           H-SPNITPAE KFRLSEVTG- LKPKQVTIWF QNRRNRKGKK NLNVE---PT
           H-APNITQAE KYKLAQATG- LQPRQVTIWF QNRRNRRAHA RRAAIVDSGL
           H-APNITQAE KYKLAEATG- LQPRQVTIWF QNRRNRRSHT RRSTT-----
           R-TANISRAE RIRLAALTK- LEPRQVTIWF QNRRNRKAKA LASKT-----
           Y-TTNITQAE KRRLAQATG- LEPRQVVIWF QNRRNRKVKS RTEAG-----
           H-AQNITQAE KRKLAELTG- LQPRQVVIWF QNRRNRKVKT QRRSV-----
           Q-TTNINKSE KLQLAKATN- LEPRQVTIWF QNRRNRKPTA QAHSN-----
```

```
H-TPNITQAE KYKLAEATG- LLPRQVTIWF QNRRNRKAAK SKNAA----A
H-TPNITQAE KFQLAEATG- LQPKQVTIWF QNRRNRKGKK GSKLA----I
H-TPNITQSE KYKLAEVTG- LQPRQVTIWF QNRRNRKAKA PPKPLKKSAK
H-AQAITIAE KQRLAAATG- LEPRQVTIWF QNRRNRKTKD GEARY---VS
HCSESVTAAE RQWLAEIIGC LNERQVMIWF QNRRHRGGSR RSSIH-----
I-TPNITQAE KFRLAEVTG- LQPKQVTIWF QNRRNRKGRK GMALK--HVA
R-AHSITMAE KHRLAAATG- LEPRQVTIWF QNRRNRKTKQ GTHKY--HAR
H-TPNITQAE KYRLAEVTG- LQPKQVTIWF QNRRNRKGRK GAKGA-----
R-TDKITQAE RDRLSEASG- VPPRSVTIWF QNRRNRPRRT TKVKT-----

ESTQPDLSPS RHESPPPSSP SRDFTLSE-- ---------- ---KKRKSYG
VSPEPELPPP PRLEAPPPPP SVAAARVGQT D--------- ---DGKDDML
---------- --VEPSPAFL TSKTTLGS-- ---------- ---PSKSPLN
-------GNE CELTPVCATP IKSS------ ---------- ---RKRKAEG
---------- ----APSTAV HTVSPSAH-- ---------- ---LARKRKM
------LEVK QEDDAAAASP PAS------- ---------- ---RKRQR--
--------TK SQKQSSSSLP LR-------- ---------- ---RKRKS--
AANDPKQGQE EIGGGGGEAA ATTKTATS-- ---------- ---ERKRKTA
TSQSDSDPLS FKNGSPPPSP MRDFTLSE-- ---------- ---KKRKSYG
SQPEPKSQTA PQQIPPATVP VPLFPLASAY LTRSPTYQLT QLTSKRKPIA
GSAKSTAAEP SASSPAADAF SRDADAAGAA AQGRA----- ---SGKRKSM
---------- --------ST LRSFSPST-- ---------- ----------
QSPTDLSPAK REATPPPSSP TRDFTLSE-- ---------- ---KKRKSYG
GGAHETIKTD LSLEFDFDAT SVTSSSIS-- ---------- ---SKRK---
KNSHARVTAR AQSPNDCFSP PRDFTLNE-- ---------- ---KKRKSYG
-EAQAEDISL RATPLLPSSP VATSPPLV-- ---------- ---GRKRKAG

VLGRSSPDCT DPDSDSPSSS LKKPR----V SS-----VCS KLSDGSSSS-
VEGEESGASS LSDWASATTV PSDSL----- -------SFD APIRPFGSSS
FVKSEIACEI DAESDFGFEY QEGAT----- -------HSE SEHDSQPMSK
LYDGETRQ-- ---------P SQRMR----- ---------- -TSTPDNSCL
SCNDAAS--P RRPSSFVT-P EKKAR----L PD-------- --MPPPPID-
--DHDEREDD SQPPPPPYEH IKRPR----- -------TTA LATPPPMFD-
---------D EMDGIVPSTP KHRRR----M SD-------- ----------
NLNSDVSDHP LYPGTLPSVL QPLNRQQEEH DQ-----HHD QSKEWTKRRM
ALGRASLDSS SYDSDSPSSL PKKPR----L PS-----AES EVSDGSASS-
GLGLSIPDVS DTESNASSCA SKKRRTARIA SDDSFNSTCT IDTTDTAFS-
ALDDERDDAP RLPSTFGHDA DKRRR----M AD------GS TQRTGSGSSA
---------- -EDGDTSSEP QSSAM----- ---------- ----------
ALGRSSSDFS DSDSDSPSSH LKKPR----L PS-----ACS DDSEGSASS-
--GRTLHAEL SLPSEL---Y QKRQR----L PN-----GSA RRISSTGSDG
ALGVASSSSS DSDSDEG--F LKKPR----L PR-----AHS GLSDASTSS-
LYDDEVGVRM PSDD-----P RQRRR----L SI-----STD LSLSP-----

YEYNDVFTQW GSPSSHSTSL SSES------ ---------- SGLSDFESPR
PAQLANSTFS TSPLSTTSSL IL-------- ---------- HASQPHMSPQ
LDAESDYESD GRSQIVTEIP TISLP----- ---------- VLPSVSESSS
SRYTNHYTSS SSSSSPSSSS SSVS------ ---------- SFDNLASFGR
RRLLSGSSNA SVGSSIDSRT ES-------- ---------- LLSGWAGSER
RRLFSSSSSG SLASSVDSGS ---------- ---------- SLLSWHRDQR
SGMSPGLSRD VTPFSISSRG RS-------- ---------- SSVDLFEAGH
LEDGRIVSSS SSSSSTSFSS SSSAAAPSGD RYTSYSSYGY IPPPHLGDPA
YEHHAAFTCW STPSSHSNSS SLDL------ ---------- SGMSDFDSPR
LDSLTSYNAW SASSNSSGSL SDD------- ---------- WAGQVNEAHH
SSLASRSSSS SLGSSHTSWD SVPRSAKARG GSGSSGTTVT SMESWAPMSY
---------- ---------- ---------- ---------- ----------
VDLINAFTPW STPSSRSTSS SSASS----- ---------- SHSDIFDSPR
SSEADSLQSW TADSSAAQQR NLSGVS---- ---------- SLDTTVDNGS
SAHTASFTAW SSPSSRSTSS SSVSS----- ---------- SPSDCFDSPG
PRHHSGSSSS SSSMTASSND ---------- ---------- SLYSWSQEAA

RPRNIFDYMR PRAMDGKAVA A-------MP RLTI------ SAPQHPHCAT
MQTQAQMTPV PQAHTQTQAQ G-------HT QLPIQVASHP PPPSIPLQSQ
DPIHNYVGAE ACEDNVLDVW SSS-----MP VTSI------ ----------
GRHGKQRNDS SDSSELEGVD P-------VG VIEF------ ----------
YGQQ--RAVS SESAATSYTS PSICSSFAST LASE-----E CSSSTRVNAQ
QPDA--SSET TFSSPLSSNS P-------AS VWHD------ ----------
AMSSGTSYDD SFSAYQSISV S-------PP RSTF------ ----------
KPRNPSGQSH SSDSSFVSSV ATSLLDNFHP NPIVMHNSLL LPPPTLSVSD
KPRNVFDYMK PRTLNVKAIT S-------MP KLTI-----P APQQHASTNA
DPNSVAGNVF RSVSHFTPDQ P-------MP EVTMTHDAAA MAAQQHTSVD
KPPG--MSLT GAAAHMSEVE L-------QP VWLA-----G SQSQGSLHGG
---------- ---------- --------SP KLSA------ ----------
RPHNLFKYIS P-KYEGRADV S-------MP HLTI-----G TPQKMAHQGL
RPSTPFDDLT SDTRLQLGYN R-------HT TLAC------ ----------
KAHNVFRLLN PLKYDARAKQ D-------MP AVTM------ -ATPSLMPYQ
ERLRNVSWDT NFSSTYDDRT T--------- ---------- ----------

ASDQKSPF-- ---------- ---------- -LYELQDSTF FDGTRLDLSG
ASMSMTPF-- ---------- ---------- -------QAP ASPATLSTAA
---------- ---------- ---------- ---------- -----LEYSS
---------- ---------- ---------- ---------- ----------
EARRKQPA-- ---------- ---------- ---------- ---SVRSEQE
---------- ---------- ---------- ---------- ----------
---------- ---------- ---------- ---------- ----------
NVFHRKSS-- --------EM GENFND---- ---HMLFRPD HCLPMLDLDD
TLGQRSPF-- ---------- ---------- -LCDIATSKV ATGANLDFSG
TSSLSSAFRL YTPETSCTSV GSSFASPKQP EELDYVFNTE TGNAQLDLAD
GLSQQQCM-- ---------- ---------- ---------- ----------
---------- ---------- ---------- ---------- ----------
APAQRSPF-- ---------- ----TS---- ---DIHAGTN SQMGRIDLSG
---------- ---------- ---------- ---------- -------FTA
CAEDRSPF-- ---------S SDAANGGMYG GPQRLQFARE AMQHEVDFGG
---------- ---------- ---------- ---------- ----------
```

```
LQLNLGGFAD DKDFRESVQM ALSMSSS-EQ GSSRSASSSS -WASTQATTD
TATNTTTRAS SLSSTTTTCM PSASTAPIVS LDQLLDLDNA ---RQCLVFS
MSYPSTSNSD SHTHGPAAVL FCGGSSQFMQ TGHSASLDDE ---GQYLVFS
GKNDLGQVST PFAMGDNVVE KEKQQLSCNN ETKEEDALTT -------MLF
QAFAQRLLPS SPAHGHREPP PAEIAHIFND GQEHLASCGL -WGSSNFLFT
---------- ------SPCK SRSTNTAAPF LGPNPVLLAP -SGETYIFQD
----GASSFD EQIVRFTNAK NSGSSSNSSS NGNSRNSSNT ----SNEMMF
LGLDAITLQR TFNVNWKVAM GGVESPSIGT GAVTPKRNVD VDVATCPGTE
LYFNLDSVAD DQSFRESVQR VLSNMSGYEA DISRNVSSSS -WSSQQTTTD
LELIEDSLTQ ALQQDLSKAF DFSSALKTPA SHTQVTLEDD -WIESVAFTP
-RQHDEHISQ SLSMMDTAYA APQSSQASSD GSPPPEPSCT -PLPGDMLLL
---------- ---------P NQAIESTTDS PMTRPSVAQE -------GFA
LQLNMGEAFD RDFRESVQRV FSG--SGFDV GSYRSVSSSS -WGSQA-VTT
SPHTEQLSVP STSIKGFERQ HPELSGEGAS TEPSPYACSE -RYEDAALLF
LQFDADAMGR DL----CESV QRVLEMNFSS FADRSMSDSS -WGSTMQSAT
---------- EDDVSEQKKG EEKDKGKGTS LIDSPVLSEV ----EMPMLF

DDG---WVDE EDFDSGFAAC HTKPIDRTLL ---------- ----GQASLT
PLDLLPRLDF DDLRLDVPTI ENWLG----- ---------- ----LPSTPG
PVDSMPRLDF ADLNLNIDAL EE-------- ---------- ----CFGLSM
SETGVTQLDF DDLGLDLDSM VIDLPS---F ---------- ----GFDFST
DDSDVPRLDF DDLGLEPADF GKEGSLLFDF AAPPPAGGAQ LWQRGPPMVG
EETNVPQLGF EELQLDPQSL NENLKLSF-- ---------- ----DGLAAG
NENGELLLDW DDLELNLDDF TGTQSLTPLT ---------- ----DNQNNG
DFNDDGWMDE DDDQAAWNGE SSTLA----- ---------- ----AGKKTL
DDG---WEDV DD-SIASHVN GGWQVQISS- ---------- ----GPTQVD
NNNDVEWVNI DEFTRAPKKQ SRPLW----- ---------- ----SMSQEE
DAYGQQLLDW DDLELDASLN GALVL----- ---------- ----SPEQLD
DQE---WL-- ---------- ---------- ---------- ----------
DDDG--WVDE DDFDASLDGR HETPVDAA-- ---------- ----VQGQNS
DAYGVAQLDW DDLCLDSHLS AAL------- ---------- ----GAAENA
DDDG--WVDE DEPSAS---- ---------- ---------- ----TTPVPI
DSRGNVQLGF ADLQLDLGYL KERLS----- ---------- ----GAAGPS

PPDHCNSNTA PGQASRQEIF QAPCVSG--- -SHPANHSQT SADENP----
VSSLHVPPIS AGAMRSAKLA LSQPLNA--- ---PDELMGR AADEGW----
ESDNTMIRVR KFEQEEVRTA EYLCSNP--- FNVTEELESR ATEEDW----
FALPQPIPWI SDSSAKTSVN NSPLAPQ--- ---PCFATPQ V-PTSTLHAA
VPSGTLHNQY PFAATSTTTL GYALDPSA-- ---SAQDRTS SRPRHFTDVA
TQPGLVNGWQ RSDVTNCTPG LAGVAAGR-- ---HDQDKES LMEANAFGLA
ILAGLDLPNS AHSTQETNDL VSIFARK--- -----LVEES FNPEEF----
RSRGKAIKHI SNKAGSQVNY DDVDATPKQE DFNFQHHHQA SSPKPR----
AMPSQEAPQT TLLPRAQSAP LDQTNNG--- ---------- ----------
QTTPTVARVN AGDKSNTTAG ISGFASG--- -SCPDQTPRP GCESPF----
LASGLSAELQ A--AGWSPGG MDLTTPRQ-- ---PSFAQSA STPVSP----
---------- -----TVPIS VHGDTPG--- ---------- ----------
MMPPAFHSVN TPMLGQTPLQ VLQPQSV--- ---PPTMPGA GADTTP----
LLSGLTTPKL R--PGTAPSA TSLMSPRKSG FRSPSLAPNA ASDPSA----
SASNNVLPQQ PDAHVLSQPT QGAMQLGHVA FTSAPVHSAA VSTAPW----
FTIAQPGGLS PDDAESEPAM GTSETSQQ-- ---SSASSSR LSPTSPLLMA

---------S ISIPFSQSAL FDS-DSFGLD QLFE------ ---------S
SLSEQLTPLW NV-------- -----PTDAH RLSD------ --------LS
------DISS IITRELTRSL DGK-EMQDLS VRLE------ ------GISS
TILNNSTVDA LEEIRRAQQI EEW-AFGTNA ILEEDT---- ---DNKGSYF
ESLNEGDFNL FAALASLPTF KDM-SPDAIL QFAASGGHDL SVDAERTRYA
LTLSGEHSCG ANTLAPSAAA GED-SMDGLF DFD------- ---------F
LAQMQQSEEW SSPVMGRLFL ARG-QEQDAI SLAD------ --------LQ
--LSGRAIET SEASEAARQL TDS-EVNILE FIFEKVHKET SGIERGRGAG
------LNTG SDNAHSQPSL FGD-DSFDLS QFFQ------ ---------S
---QSTAQET PTNGGSCNQA SQL-SLEDMM FLSQ------ ---------L
ATISQTILAW PESTTDASAF FASFGPEAFA DLLP------ --------AD
---------- ---------- ----APLEFA FFMD------ ----------
---------- -DSSSFCPPS FNN-EPFDLA RLLE------ --------LA
HAIGAAITDW PSAPEIASQM AMGLSTESAR DMFV------ ---------A
--ANPAGFAS NGNGSSDSAL SNG-EPIDLH NFSE------ ----------
TLLNQATISV PNELRRASVN GKW-TFDASK MLEG------ ----AGDEWF

ASIPAHLPST LSHSQQQGFG QMPFV----- ---------- --DPNMQSFE
LRIDGTMLRR DSVMKAERMA PPPSA----- ---------- ----------
TRSNMEEAHR RLFQSYPVSP MTPCL----- ---------- ----------
LNLSGLRSAG TDTPSSTSEA STDGN----- ---------- GSSVVTPNEL
QSLPTLGSSP SSSTPREKDI VTPPS----- ---------R VADANSPEDF
SAPDAANGSS SDFDFSFGLD LPFCF----- ---------- -----SPKQL
STQNSGPSAS TSTQTSPGSS TPRSL----- ---------- ----------
STTFSSSSSS SSSISTPGEG YTPGW----- ---------- -WMNCDLVVS
TSVPTHMPSI MTFPQQHGLF EVPLP----- ---------- --QVHTQELG
VDSYPHNIAP TQVAQSTYHE GQPCLDLRID MFDIDRLFQL LEAENYQYQA
ADPKAQAPQE IHVPVHVDIG PDTSF----- ---------- ----------
---------- ---------- ---------- ---------- ----------
AAAPAHLPTS SPFASQPQQP LPTED----- ---------- ----------
IEVPDAMDIS EDAKPVMVLS VPIPS----- ---------- ----------
--VGSHAPAP ASYAPYQQTS PQQPV----- ---------P TASTTPVVNA
VKLDVPTPST SSSSPSSVAS PQTPS----- ---------- ----------

EIEQDLDLDM TDIQEFLGGD IFASSLPGSQ QSNGC----V GSADANVQGS
---------- ---------- ---------- ---------P LTTQSYPLPL
---------- ---------- ---------- ---------- ---PPYSSLS
-----FDFSL QGL-YINQIM VANLATFEEP EDGT-----G TSKSISHHLP
----AFTFDF AEL-GLDRSF AEIRKSYRVA DEEAKMYSIS EVEDEEGGGA
GLPLAFDFAR QAFSDVEAEM YASYSSCEMS GSDT-----D GDAEVELHGT
-----FNFDF DNL-GLEDAM TYNNESIG-- ---------Q QINSIDLHAM
QTPPPSTLEN GSVPNVDDYL SFPGESDEGK QAIAE----E MDFNLASLGI
EIEKPLDMDL DDIQSILDSN IFASSLPSSQ QSNGGIA--P QNSSAQGCNA
QGDLQYALNF DDSGDLVQSI DSTNENDSGF DSQP-----N LQIRIFRLAS
```

```
----SFAAGL ESWAACPTSL EHVIEQWKGF ---------V PSATPEELAS
---------- ---------- ---------- ---------- ---------A
---TNLDIEM TDIQSLFDRD LFDASSLP-- ---------G SQQSNAGDGA
---------- -------GIL VKNGSSSG-- ---------A IQLTPESFAS
NAFLDFEVEM ADLDDLLSSF VQPAVP---- ---------A PTAAAEVGAG
RDLFNFDLES LQMHDIVQAN IKTYDCT--- ---------Y NESTPTPTPT

NGGASIGEGI LHMDFDPYSN SFSLA----- ---------- ----------
SARWNHDAIA PNVLTWDLSR GISSTLVP-- ---------- ----------
QGYVAPSTQS FEPMNITSPS LMECSH---- ---------- ----------
LETMAANESS FDVAMATARD TMHC------ ---------- ----------
TSGWETGPEE MKGRGTKRSS SGTIISERHT SSETSSGPWA IPARPVDLHG
TDRRAAKATA ATSPSIQGPH ATSVAF---- ---------- ----------
EELLKYEPLT TDMLGLIFSP PASASPIANV DTASLTLLN- ----------
NEAWEENRKM MAMTMSMAPP FFHGLPSDQT QMVEVEMTDG GFW-------
GTGAEMGEGV FQMNFDLDSN SFGYV----- ---------- ----------
LSLLEFHPAT LSSRDVLARF SFPSPFSIDS HDTLPSIYIY TRHIPAPHV-
SSAMDIDDSQ MNELGELRRH SEQALFYEES QQPMLLA--- ----------
LGLPSSTDVQ MHAN------ ---------- ---------- ----------
LQGNAAAEAQ FCMNFDMSSN AFSMV----- ---------- ----------
LESWTHDASS LEYI------ ---------- ---------- ----------
VQVTQQQPAE FYLNFDLSPD MFRAA----- ---------- ----------
AHQFCDAAKE PCQLSGLKPE DDDISMEISS Q--------- ----------

---------- --
---------- --
---------- --
---------- --
QRQAGGRFFA S-
---------- --
---------- --
---------- --
---------- --
---------- --
---------- --
---------- --
---------- --
---------- --
---------- --
---------- --
```

Figure S2 Alignment of bW (HD2) protein sequences. The bW protein sequence from *Ustilago maydis* (UMAG_00578) was used as a reference sequence to determine orthologs in all 16 Ustilaginomycotina genomes. Then, all orthologous sequences were used for the alignment using the MUSCLE algorithm performed in MEGA-X. Species abbreviations are as follows: Acain, *Acaromyces ingoldii*; Cergu, *Ceraceosorus guamensis*; Exova, *Exobasidium vaccinii*; Jamro, *Jaminaea rosea*; Malgl, *Malassezia globosa*; Malsy, *Malassezia sympodialis*; Meimi, *Meira miltonrushii*; Psean, *Pseudozyma antarctica*; Psehu, *Pseudozyma hubeiensis*; Psegl, *Pseudomicrostroma glucosiphilum*; Spore, *Sporisorium reilianum*; Tescy, *Testicularia cyperi*; Tilan, *Tilletiaria anomala*; Tilwa, *Tilletiopsis washingtonensis*; Ustma, *Ustilago maydis*; Viopa, *Violaceomyces palustris*.

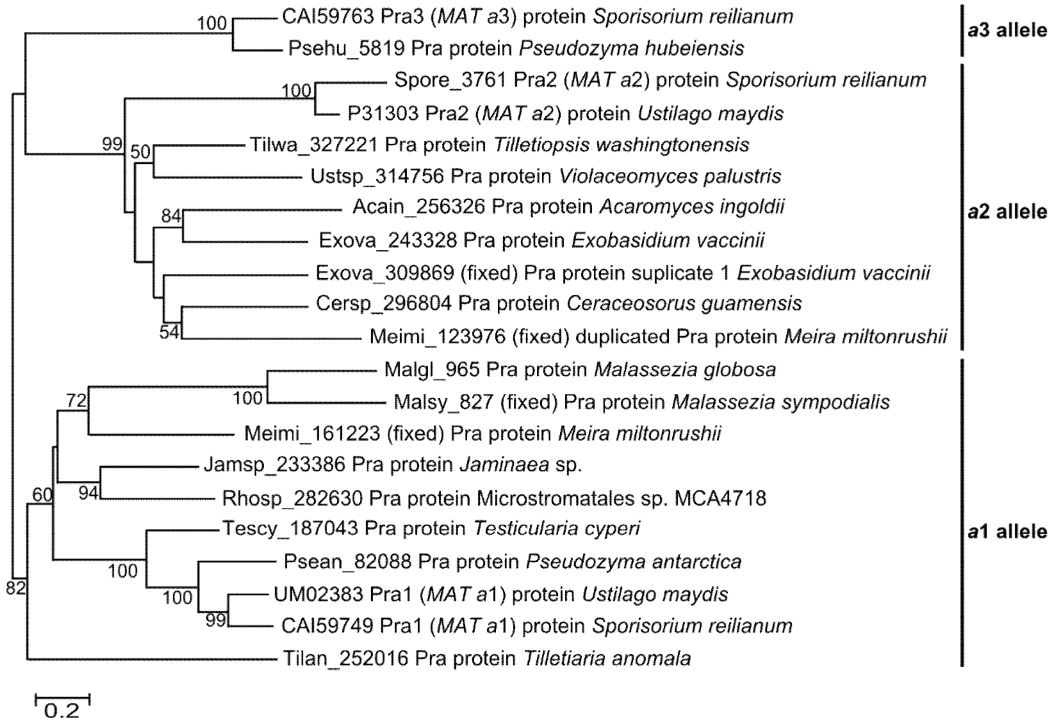

Figure S3 Gene phylogeny of pheromone receptors Pra1. The pheromone receptor protein sequence from *Ustilago maydis* (UMAG_02383) was used as a reference sequence to determine orthologs in all 16 Ustilaginomycotina genomes. All orthologous sequences, plus the ones from each *MAT a* allele in *Ustilago maydis* and *Sporisorium reilianum*, were retrieved for the analyses. The protein sequences were aligned using the MUSCLE algorithm, and then used for phylogenetic reconstruction through the Neighbor-joining method with JTT as a substitution model and a gamma distribution as a rate heterogeneity among sites. The 1000 replicates of bootstrapping were used as an indicator for node supports. Allele types of pheromone receptors are classified based on monophyletic relationships with reference sequences from *U. maydis* and *S. reilianum*: UMAG_02383 for the *a1* allele in *U. maydis*, CAI59749 for the *a1* allele in *S. reilianum*, P31303 for the *a2* allele in *U. maydis*, Spore_3761 for the *a2* allele in *S. reilianum*, and CAI59763 for the *a3* allele in *S. reilianum*. Species abbreviations are as follows: Acain, *Acaromyces ingoldii*; Cersp, *Ceraceosorus guamensis*; Exova, *Exobasidium vaccinii*; Jamsp, *Jaminaea rosea*; Malgl, *Malassezia globosa*; Malsy, *Malassezia sympodialis*; Meimi, *Meira miltonrushii*; Psean, *Pseudozyma antarctica*; Psehu, *Pseudozyma hubeiensis*; Rhodsp, *Pseudomicrostroma glucosiphilum*; Spore, *Sporisorium reilianum*; Tescy, *Testicularia cyperi*; Tilan, *Tilletiaria anomala*; Tilwa, *Tilletiopsis washingtonensis*; Ustma, *Ustilago maydis*; Viopa, *Violaceomyces palustris*. Bar: 0.2 substitution/site.

Table S1 A list of known fungal dimorphism genes from *Ustilago maydis* literature

Categories	Gene name	Function	References
Receptors	*Pra1*	Pheromone receptor	[1]
	Msb2	Transmembrane mucin, multicopy suppressor of a budding defect	[2]
	Sho1	Osmosensor transmembrane protein	[2]
	Ump2	Ammonium transporter	[3]
cAMP/PKA pathway	*Gpa3*	G protein alpha subunit	[4]
	Bpp1	G protein beta subunit	[5]
	Uac1	Adenylate cyclase	[6]
	Ubc1	Regulatory subunit of cAMP-dependent protein kinase A	[6,7]
	Adr1	Catalytic subunit of cAMP-dependent protein kinase A	[8]
	Uka1	cAMP-dependent protein kinase A	[8]
	Umpde1/2	Phosphodiesterases	[9]
	Ucn1	Antagonist phosphatase of PKA	[10]
MAPK pathway	*Ubc2*	Pheromone-responsive SH3 domain protein	[11,12]
	Kpp2/*Ubc3*	MAP kinase	[13,14]
	Kpp4/Ubc4	MAP kinase kinase kinase	[15,16]
	Kpp6	MAP kinase	[17]
	Fuz7/Ubc5	MAP kinase kinase	[16,18]
	Rok1	Dual specificity protein phosphatase	[19]
	Crk1	MAP kinase	[20]
GTPase-mediated signaling	*Ras1, Ras2*	*Ras* family GTPase	[21,22]
	Sql2	Cdc25-like guanyl nucleotide exchange factor	[22]
	Rho1	*Rho* family GTPase	[23]
	Pdc1	14-3-3 homolog	[24]
	Cla4	PAK family kinase	[25]
	Rac1	*Rho* family GTPase	[26]
Transcriptional regulator	*Biz1*	*b* locus-dependent Zn finger transcription factor	[27]
	Hap2	CCAAT-box binding protein	[28]
	Rop1	High-mobility-group (HMG) domain transcription factor	[29]
	Prf1	Pheromone response factor functioning as a transcription factor	[30,31]
	Rbf1	*b* locus-dependent Zn finger transcription factor	[32]
	Cib1	*b* locus-dependent Zn finger transcription factor	[33]
	Gcn5	Histone acetyltransferase	[34]
	Hos2	Histone deacetylase	[35]
	Tup1	General transcriptional repressor	[36]
	Pac2	WOPR family transcriptional repressor	[36]
	Ros1	WOPR family transcriptional regulator	[37]
	Nit2	GATA transcription factor responsive to low nitrogen	[38]

	Med1	Transcription factor	[39]
Other downstream molecular players	*Hgl1*	Putative regulatory protein	[40]
	Rak1	Seven-WD40 repeat motif protein	[41]
	Myo5	Class V myosin	[42]
	Kin1, Kin3	Kinesin-1 and 3 required for hyphal growth	[43]
	Rrm4, Khd4	RNA-binding protein for filamentous growth	[44,45]
	Clb2	B-type cyclin 2	[46]
	Chs5	Chitin synthase V	[47]
	Chs7	Chitin synthase	[47]
	Mcs1	Myosin chitin synthase 1	[47]
	Clp1	Function in nuclei distribution during cell division in dikaryon	[33]
	Yup1	*t*-SNARE protein for vesicular cycling	[48]
	Sep3	Septin 3 as an effector of cAMP/PKA pathway	[49]
	Tea1, Tea4	Cell end markers	[50,51]

References

1. Bölker, M.; Urban, M.; Kahmann, R. The a mating type locus of *Ustilago maydis* specifies cell signaling components. *Cell* **1992**, *68*, 441–450.

2. Lanver, D.; Mendoza-Mendoza, A.; Brachmann, A.; Kahmann, R. Sho1 and Msb2-related proteins regulate appressorium development in the smut fungus *Ustilago maydis*. *Plant Cell* **2010**, *22*, 2085–2101.

3. Smith, D.G.; Garcia-Pedrajas, M.D.; Gold, S.E.; Perlin, M.H. Isolation and characterization from pathogenic fungi of genes encoding ammonium permeases and their roles in dimorphism. *Mol. Microbiol.* **2003**, *50*, 259–275.

4. Regenfelder, E.; Spellig, T.; Hartmann, A.; Lauenstein, S.; Bölker, M.; Kahmann, R. G proteins in *Ustilago maydis*: transmission of multiple signals? *EMBO J.* **1997**, *16*, 1934–1942.

5. Müller, P.; Leibbrandt, A.; Teunissen, H.; Cubasch, S.; Aichinger, C.; Kahmann, R. The Gβ-subunit-encoding gene bpp1 controls cyclic-AMP signaling in *Ustilago maydis*. *Eukaryot. Cell* **2004**, *3*, 806–814.

6. Gold, S.; Duncan, G.; Barrett, K.; Kronstad, J. cAMP regulates morphogenesis in the fungal pathogen *Ustilago maydis*. *Genes Dev.* **1994**, *8*, 2805–2816.

7. Gold, S.E.; Brogdon, S.M.; Mayorga, M.E.; Kronstad, J.W. The *Ustilago maydis* regulatory subunit of a cAMP-dependent protein kinase is required for gall formation in maize. *Plant Cell* **1997**, *9*, 1585–1594.

8. Dürrenberger, F.; Wong, K.; Kronstad, J.W. Identification of a cAMP-dependent protein kinase catalytic subunit required for virulence and morphogenesis in *Ustilago maydis*. *Proc. Natl. Acad. Sci.* **1998**, *95*, 5684–5689.

9. Agarwal, C.; Aulakh, K.B.; Edelen, K.; Cooper, M.; Wallen, R.M.; Adams, S.; Schultz, D.J.; Perlin, M.H. *Ustilago maydis* phosphodiesterases play a role in the dimorphic switch and in pathogenicity. *Microbiology* **2013**, *159*, 857–868, doi:10.1099/mic.0.061234-0.

10. Egan, J.D.; García-pedrajas, M.D.; Andrews, D.L.; Gold, S.E. Calcineurin Is an Antagonist to PKA Protein Phosphorylation Required for Postmating Filamentation and Virulence , While PP2A Is Required for Viability in *Ustilago maydis*. *Mol. Plant-Microbe Interact.* **2009**, *22*, 1293–1301.

11. Klosterman, S.J.; Martinez-Espinoza, A.D.; Andrews, D.L.; Seay, J.R.; Gold, S.E. Ubc2, an ortholog of the yeast

Ste50p adaptor, possesses a basidiomycete-specific carboxy terminal extension essential for pathogenicity independent of pheromone response. *Mol. plant-microbe Interact.* **2008**, *21*, 110–121.

12. Mayorga, M.E.; Gold, S.E. The ubc2 gene of *Ustilago maydis* encodes a putative novel adaptor protein required for filamentous growth, pheromone response and virulence. *Mol. Microbiol.* **2001**, *41*, 1365–1379.

13. Müller, P.; Aichinger, C.; Feldbrügge, M.; Kahmann, R. The MAP kinase kpp2 regulates mating and pathogenic development in *Ustilago maydis*. *Mol. Microbiol.* **1999**, *34*, 1007–1017.

14. Mayorga, M.E.; Gold, S.E. A MAP kinase encoded by the ubc3 gene of *Ustilago maydis* is required for filamentous growth and full virulence. *Mol. Microbiol.* **1999**, *34*, 485–497, doi:10.1046/j.1365-2958.1999.01610.x.

15. Müller, P.; Weinzierl, G.; Brachmann, A.; Feldbrügge, M.; Kahmann, R. Mating and pathogenic development of the smut fungus *Ustilago maydis* are regulated by one mitogen-activated protein kinase cascade. *Eukaryot. Cell* **2003**, *2*, 1187–1199.

16. Andrews, D.L.; Egan, J.D.; Mayorga, M.E.; Gold, S.E. The *Ustilago maydis* ubc4 and ubc5 genes encode members of a MAP kinase cascade required for filamentous growth. *Mol. plant-microbe Interact.* **2000**, *13*, 781–786.

17. Brachmann, A.; Schirawski, J.; Müller, P.; Kahmann, R. An unusual MAP kinase is required for efficient penetration of the plant surface by *Ustilago maydis*. *EMBO J.* **2003**, *22*, 2199–2210.

18. Banuett, F.; Herskowitz, I. Identification of fuz7, a *Ustilago maydis* MEK/MAPKK homolog required for a-locus-dependent and-independent steps in the fungal life cycle. *Genes Dev.* **1994**, *8*, 1367–1378.

19. Di Stasio, M.; Brefort, T.; Mendoza-Mendoza, A.; Münch, K.; Kahmann, R. The dual specificity phosphatase Rok1 negatively regulates mating and pathogenicity in *Ustilago maydis*. *Mol. Microbiol.* **2009**, *73*, 73–88.

20. Garrido, E.; Pérez-Martín, J. The crk1 gene encodes an ime2-related protein that is required for morphogenesis in the plant pathogen *Ustilago maydis*. *Mol. Microbiol.* **2003**, *47*, 729–743, doi:10.1046/j.1365-2958.2003.03323.x.

21. Lee, N.; Kronstad, J.W. ras2 controls morphogenesis, pheromone response, and pathogenicity in the fungal pathogen *Ustilago maydis*. *Eukaryot. Cell* **2002**, *1*, 954–966, doi:10.1128/EC.1.6.954-966.2002.

22. Müller, P.; Katzenberger, D.; Loubradou, G.; Kahmann, R. Guanyl Nucleotide Exchange Factor Sql2 and Ras2 Regulate Filamentous Growth in *Ustilago maydis*. **2003**, *2*, 609–617, doi:10.1128/EC.2.3.609.

23. Pham, C.D.; Yu, Z.; Bolker, M.; Gold, S.E.; Perlin, M.H. Ustilago maydis Rho1 and 14-3-3 Homologues Participate in Pathways Controlling Cell Separation and Cell Polarity. *Eukaryot. Cell* **2009**, *8*, 977–989, doi:10.1128/EC.00009-09.

24. García-pedrajas, M.D.; Nadal, M.; Bölker, M.; Gold, S.E.; Perlin, M.H. Sending mixed signals : Redundancy vs . uniqueness of signaling components in the plant pathogen , *Ustilago maydis*. *Fungal Genet. Biol.* **2008**, *45*, S22–S30, doi:10.1016/j.fgb.2008.04.007.

25. Leveleki, L.; Mahlert, M.; Sandrock, B.; Bölker, M. The PAK family kinase Cla4 is required for budding and morphogenesis in *Ustilago maydis*. *Mol. Microbiol.* **2004**, *54*, 396–406, doi:10.1111/j.1365-2958.2004.04296.x.

26. Mahlert, M.; Leveleki, L.; Hlubek, A.; Sandrock, B.; Bölker, M. Rac1 and Cdc42 regulate hyphal growth and cytokinesis in the dimorphic fungus *Ustilago maydis*. *Mol. Microbiol.* **2006**, *59*, 567–578.

27. Flor-Parra, I.; Vranes, M.; Kämper, J.; Pérez-Martín, J. Biz1, a zinc finger protein required for plant invasion by *Ustilago maydis*, regulates the levels of a mitotic cyclin. *Plant Cell* **2006**, *18*, 2369–2387.

28. Mendoza-Mendoza, A.; Eskova, A.; Weise, C.; Czajkowski, R.; Kahmann, R. Hap2 regulates the pheromone response transcription factor prf1 in *Ustilago maydis*. *Mol. Microbiol.* **2009**, *72*, 683–698.

29. Brefort, T.; Müller, P.; Kahmann, R. The high-mobility-group domain transcription factor Rop1 is a direct regulator of prf1 in *Ustilago maydis*. *Eukaryot. Cell* **2005**, *4*, 379–391.

30. Hartmann, H.A.; Kahmann, R.; Bolker, M. The pheromone response factor coordinates filamentous growth and pathogenicity in *Ustilago maydis*. *Embo J* **1996**, *15*, 1632–1641.

31. Hartmann, H.A.; Krüger, J.; Lottspeich, F.; Kahmann, R. Environmental signals controlling sexual development of the corn smut fungus *Ustilago maydis* through the transcriptional regulator Prf1. *Plant Cell* **1999**, *11*, 1293–1305.

32. Heimel, K.; Scherer, M.; Vranes, M.; Wahl, R.; Pothiratana, C.; Schuler, D.; Vincon, V.; Finkernagel, F.; Flor-Parra, I.; Kämper, J. The Transcription Factor Rbf1 Is the Master Regulator for *b*-Mating Type Controlled Pathogenic Development in *Ustilago maydis*. *PLOS Pathog.* **2010**, *6*, e1001035.

33. Heimel, K.; Scherer, M.; Schuler, D.; Kämper, J. The *Ustilago maydis* Clp1 protein orchestrates pheromone and b-dependent signaling pathways to coordinate the cell cycle and pathogenic development. *Plant Cell* **2010**, *22*, 2908–22, doi:10.1105/tpc.110.076265.

34. González-prieto, J.M.; Rosas-quijano, R.; Domínguez, A.; Ruiz-herrera, J. The UmGcn5 gene encoding histone acetyltransferase from *Ustilago maydis* is involved in dimorphism and virulence. *Fungal Genet. Biol.* **2014**, *71*, 86–95, doi:10.1016/j.fgb.2014.09.002.

35. Elías-Villalobos, A.; Fernández-Alvarez, A.; Moreno-Sánchez, I. The Hos2 Histone Deacetylase Controls *Ustilago maydis* Virulence through Direct Regulation of Mating-Type Genes. *PLoS Pathog.* **2015**, *11*, e1005134, doi:10.1371/journal.ppat.1005134.

36. Elías-Villalobos, A.; Fernández-Álvarez, A.; Ibeas, J.I. The general transcriptional repressor Tup1 is required for dimorphism and virulence in a fungal plant pathogen. *PLoS Pathog.* **2011**, *7*, e1002235.

37. Tollot, M.; Assmann, D.; Becker, C.; Altmüller, J. The WOPR Protein Ros1 Is a Master Regulator of Sporogenesis and Late Effector Gene Expression in the Maize Pathogen *Ustilago maydis*. *PLoS Pathog.* **2016**, *12*, e1005697, doi:10.1371/journal.ppat.1005697.

38. Horst, R.J.; Zeh, C.; Saur, A.; Sonnewald, S.; Sonnewald, U.; Voll, L.M. The *Ustilago maydis* Nit2 Homolog Regulates Nitrogen Utilization and Is Required for Efficient Induction of Filamentous Growth. *Eukaryot. Cell* **2012**, *11*, 368–380, doi:10.1128/EC.05191-11.

39. Chacko, N.; Gold, S. Deletion of the *Ustilago maydis* ortholog of the Aspergillus sporulation regulator medA affects mating and virulence through pheromone response. *Fungal Genet. Biol.* **2012**, *49*, 426–432, doi:10.1016/j.fgb.2012.04.002.

40. Dürrenberger, F.; Laidlaw, R.D.; Kronstad, J.W. The hgl1 gene is required for dimorphism and teliospore formation in the fungal pathogen *Ustilago maydis*. *Mol. Microbiol.* **2001**, *41*, 337–48, doi:mmi2528 [pii].

41. Wang, L.; Berndt, P.; Xia, X.; Kahnt, J.; Kahmann, R. A seven-WD40 protein related to human RACK1 regulates mating and virulence in *Ustilago maydis*. *Mol. Microbiol.* **2011**, *81*, 1484–1498, doi:10.1111/j.1365-2958.2011.07783.x.

42. Weber, I.; Gruber, C.; Steinberg, G. A class-V myosin required for mating, hyphal growth, and pathogenicity in the dimorphic plant pathogen *Ustilago maydis*. *Plant Cell* **2003**, *15*, 2826–2842.

43. Schuchardt, I.; Aßmann, D.; Thines, E.; Schuberth, C.; Steinberg, G. Myosin-V, Kinesin-1, and Kinesin-3 cooperate in hyphal growth of the fungus *Ustilago maydis*. *Mol. Biol. Cell* **2005**, *16*, 5191–5201.

44. Becht, P.; Vollmeister, E.; Feldbrügge, M. Role for RNA-Binding Proteins Implicated in Pathogenic Development of *Ustilago maydis*. *Eukaryot. Cell* **2005**, *4*, 121–133, doi:10.1128/EC.4.1.121.

45. Vollmeister, E.; Haag, C.; Zarnack, K.; Baumann, S.; König, J.; Mannhaupt, G.; Feldbrügge, M. Tandem KH

domains of Khd4 recognize AUACCC and are essential for regulation of morphology as well as pathogenicity in *Ustilago maydis*. *RNA* **2009**, *15*, 2206–2218, doi:10.1261/rna.1817609.

46. García-Muse, T.; Steinberg, G.; Pérez-Martín, J. Characterization of B-type cyclins in the smut fungus *Ustilago maydis*: roles in morphogenesis and pathogenicity. *J. Cell Sci.* **2004**, *117*, 487–506.

47. Weber, I.; Thines, E.; Steinberg, G. Polar Localizing Class V Myosin Chitin Synthases Are Essential during Early Plant Infection in the Plant Pathogenic Fungus *Ustilago maydis*. *Plant Cell* **2006**, *18*, 225–242, doi:10.1105/tpc.105.037341.1.

48. Wedlich-Söldner, R.; Bölker, M.; Kahmann, R.; Steinberg, G. A putative endosomal t-SNARE links exo-and endocytosis in the phytopathogenic fungus *Ustilago maydis*. *EMBO J.* **2000**, *19*, 1974–1986.

49. Boyce, K.J.; Chang, H.; D'Souza, C. a.; Kronstad, J.W. An *Ustilago maydis* septin is required for filamentous growth in culture and for full symptom development on maize. *Eukaryot. Cell* **2005**, *4*, 2044–2056, doi:10.1128/EC.4.12.2044-2056.2005.

50. Valinluck, M.; Woraratanadharm, T.; Lu, C. yu; Quintanilla, R.H.; Banuett, F. The cell end marker Tea4 regulates morphogenesis and pathogenicity in the basidiomycete fungus *Ustilago maydis*. *Fungal Genet. Biol.* **2014**, *66*, 54–68, doi:10.1016/j.fgb.2014.02.010.

51. Woraratanadharm, T.; Kmosek, S.; Banuett, F. UmTea1, a Kelch and BAR domain-containing protein, acts at the cell cortex to regulate cell morphogenesis in the dimorphic fungus *Ustilago maydis*. *Fungal Genet. Biol.* **2018**, *121*, 10–28, doi:10.1016/j.fgb.2018.09.002.
